# The Antenatal Corticosteroids Trial (ACT)’s explanations for neonatal mortality - a secondary analysis

**DOI:** 10.1186/s12978-016-0175-3

**Published:** 2016-05-24

**Authors:** Fernando Althabe, Vanessa Thorsten, Karen Klein, Elizabeth M. McClure, Patricia L. Hibberd, Robert L. Goldenberg, Waldemar A. Carlo, Ana Garces, Archana Patel, Omrana Pasha, Elwyn Chomba, Nancy F. Krebs, Shivaprasad Goudar, Richard J. Derman, Fabian Esamai, Edward A. Liechty, Nellie I. Hansen, Sreelatha Meleth, Dennis D. Wallace, Marion Koso-Thomas, Alan H. Jobe, Pierre M. Buekens, José M. Belizán

**Affiliations:** Institute for Clinical Effectiveness and Health Policy (IECS), Buenos Aires, Argentina; RTI International, Durham, NC USA; Massachusetts General Hospital, Boston, MA USA; Department of Obstetrics and Gynecology, Columbia University, New York, NY USA; University of Alabama at Birmingham, Birmingham, AL USA; Fundación para la Alimentación y Nutrición de Centro América y Panamá, Guatemala City, Guatemala; Lata Medical Research Foundation, Indira Gandhi Government Medical College, Nagpur, India; Department of Community Health Sciences, Aga Khan University, Karachi, Pakistan; University Teaching Hospital, Lusaka, Zambia; University of Colorado School of Medicine, Denver, CO USA; KLE University’s Jawaharlal Nehru Medical College, Belgaum, Karnataka India; Christiana Health Care, Newark, DE USA; Moi University School of Medicine, Eldoret, Kenya; School of Medicine, Indiana University, Indianapolis, IN USA; Eunice Kennedy Shriver National Institute of Child Health and Human Development, Bethesda, MD USA; Cincinnati Children’s Hospital, Cincinnati, OH USA; Tulane School of Public Health & Tropical Medicine, New Orleans, LA USA

## Abstract

**Background:**

The Antenatal Corticosteroid Trial assessed the feasibility, effectiveness, and safety of a multifaceted intervention to increase the use of antenatal corticosteroids (ACS) in mothers at risk of preterm birth at all levels of care in low and middle-income countries. The intervention effectively increased the use of ACS but was associated with an overall increase in neonatal deaths. We aimed to explore plausible pathways through which this intervention increased neonatal mortality.

**Methods:**

We conducted a series of secondary analyses to assess whether ACS or other components of the multifaceted intervention that might have affected the quality of care contributed to the increased mortality observed: 1) we compared the proportion of neonatal deaths receiving ACS between the intervention and control groups; 2) we compared the antenatal and delivery care process in all births between groups; 3) we compared the rates of possible severe bacterial infection between groups; and 4) we compared the frequency of factors related to ACS administration or maternal high risk conditions at administration between the babies who died and those who survived 28 days among all births in the intervention group identified as high risk for preterm birth and received ACS.

**Results:**

The ACS exposure among the infants who died up to 28 days was 29 % in the intervention group compared to 6 % in controls. No substantial differences were observed in antenatal and delivery care process between groups. The risk of pSBI plus neonatal death was significantly increased in intervention clusters compared to controls (2.4 % vs. 2.0 %, adjusted RR 1.17, 95 % CI 1.04–1.30, *p* = 0.008], primarily for infants with birth weight at or above the 25^th^ percentile. Regarding factors related to ACS administration, term infants who died were more likely to have mothers who received ACS within 7 days of delivery compared to those who survived 28 days (26.5 % vs 17.9 %, *p* = 0.014), and their mothers were more likely to have been identified as high risk for hypertension and less likely for signs of preterm labor.

**Conclusions:**

These results suggest that ACS more than other components of the intervention may have contributed to the overall increased neonatal mortality. ACS may have also been involved in the observed increased risk of neonatal infection and death. Further trials are urgently needed to clarify the effectiveness and safety of ACS on neonatal health in low resource settings.

## Background

Preterm birth remains a leading cause of child mortality and morbidity [1]. To reduce neonatal mortality associated with preterm birth, antenatal corticosteroids (ACS) for pregnant women at high risk of preterm delivery is among the most effective hospital-based interventions in high resource settings [1–7]. Currently, less than 10 % and less than 50 % of women at risk of preterm delivery in low income countries and middle income countries, respectively, receive ACS [5, 8]. Scaling up ACS has been a priority for some international health organizations [9, 10]. To that purpose, the *Eunice Kennedy Shriver* National Institute of Child Health and Human Development (NICHD)’s Global Network for Women and Children’s Health Research Antenatal Corticosteroids Trial (ACT) [11, 12] assessed the feasibility, effectiveness, and safety of a complex intervention to increase the use of ACS at all levels of care at seven study sites in low and middle-income countries (LMIC) (Argentina, Guatemala, Kenya, Zambia, Pakistan and India [2 sites]). Because the gestational age data in those settings was unreliable, we elected to define the target group as those pregnancies delivering an infant at a weight below the site-specific 5^th^ percentile.

Overall, the intervention effectively increased the use of ACS among women who delivered infants with a birthweight below the 5^th^ percentile. Forty-five percent of <5^th^ percentile births in the intervention group compared to 10 % in the control group received at least one dose of ACS (*p* < 0.0001). Of all women who received antenatal corticosteroids in the intervention group, 976 (16 %) of 6109 had delivered a less-than-5th-percentile infant. However, the intervention did not significantly reduce neonatal mortality for infants with birthweight <5^th^ percentile and was associated with an overall increase in neonatal deaths by 3.5 per 1000 livebirths in the intervention compared to the control group [12]. This harmful effect on neonatal mortality was observed among infants with a birthweight greater than the 25th percentile. The intervention was also associated with a significant increase of suspected infection in the women (2.5 % intervention vs. 1.7 % in controls, *p* < 0.0001).

The ACT results raised questions about pathways through which the intervention may have increased neonatal mortality in the general population of the intervention group. The trial was pragmatic in design, with limited data collection beyond study outcomes. The intervention was multifaceted, including training on identification of women at risk for preterm birth as well as ACS administration, the ability to identify the causal pathways of the increased mortality is limited. Nevertheless, because ACT is the largest trial of ACS in LMICs to date and because of the unanticipated results, we conducted a series of post-hoc secondary analyses to explore the trial outcomes further.

The aim of these secondary analyses was to explore plausible pathways through which the multifaceted intervention might increase neonatal mortality in the overall populations of the intervention group compared to the control group.

## Methods

### Study design and participants

ACT was an 18-month, two-arm, parallel, cluster-randomised trial to assess the feasibility, effectiveness, and safety of a multifaceted intervention designed to increase the use of antenatal corticosteroids at all levels of health care in low-income and middle-income countries. The trial methods and results are described in detail elsewhere [11, 12]. Briefly, we randomly assigned rural and semi-urban clusters within six countries (Argentina, Guatemala, Kenya, Zambia, Pakistan and India [2 sites]) to standard care or a multifaceted intervention including components to improve identification of women at risk of preterm birth, referral for care, and to facilitate appropriate use of antenatal corticosteroids. The primary outcome was 28-day neonatal mortality among infants less than the 5th percentile for birthweight (defined by site-specific data as a proxy for preterm birth). Additionally, use of antenatal corticosteroids, neonatal and perinatal mortality, and suspected maternal infection were measured for all births, irrespective of birthweight.

The outcome data were collected independently by trained Registry Administrators in a prospective maternal and newborn health (MNH) registry [13, 14], which enrolled and collected outcomes for all pregnant women residing within the study clusters, defined geographic areas which included health facilities. In addition, in the ACT intervention clusters, process data were collected on the use of ACS and the characteristics of the eligible women. The trial period included births between October 2011 and March 2014, depending on each site’s 18-month enrollment period, with most occurring in 2012 and 2013. Additionally, we included data collected during the pretrial period for births occurring mainly in 2010, although the pretrial period included some births in 2011 and 2012 in four clusters in Belgaum that were added in 2011.

#### Research questions

1. **Were ACS a direct cause of the increased mortality in the intervention group?** The multifaceted intervention effectively increased the ACS use four-fold in women who delivered <5th-percentile for birthweight infants (45 % vs 10 %) and six-fold among all women with livebirths (12 % vs 2 %), in intervention compared to control clusters [12]. However other components of the multifaceted intervention could have played a role in the observed effects. To strengthen the hypothesis of ACS as the main cause, an increased use of ACS should be observed among the neonatal deaths in the intervention group compared to the control group as well. To answer this question we compared the proportion of neonatal deaths receiving ACS between the intervention and control groups among all neonatal deaths and in the deaths of babies ≥25^th^ percentile for birthweight, the group among whom the increase in mortality was concentrated.
2. **Did the ACT intervention affect the quality of care in the intervention compared to the control group?** An explanation of the increased mortality could be that the intervention affected the quality of perinatal care and thereby increased neonatal mortality [15, 16]. We hypothesized that the harmful effects could have been mediated or confounded by aspects of care other than ACS, namely, the process of antenatal, obstetric and neonatal care. To answer this we compared the antenatal and delivery care processes between the intervention and control groups, taking into consideration pre-trial imbalances. We have focused on detecting potential clinically relevant differences in the process of care rates, rather than statistical differences. With these large numbers, small and clinically not important differences would appear as statistically significant, thus we have not conducted statistical tests for the analysis answering this question.
3. **Did the intervention increase the risk of neonatal severe infection in the intervention compared to the control group?** One hypothesis was that infection was the pathway by which neonatal mortality increased, based on the known immunosupresor effect of corticosteroids [17]. As reported in the primary paper, suspected maternal infection was higher in the ACT intervention arm [3 %] compared to the control arm [2 %]) [12]. Data on confirmed maternal or neonatal infection were not collected for the trial. However, clinical symptoms data were used to define neonatal possible severe bacterial infection (pSBI) based on the World Health Organization Young Infants Clinical Signs Study criteria [18]. An infant with any of the following was defined as having pSBI: breathing difficulty, feeding problems (i.e., stopped suckling or feeding), high fever (>38 °C), hypothermia (<35 °C), convulsions, and bleeding or pus-like discharge from umbilicus. The goal of this analysis was to compare pSBI rates, and pSBI plus death rates, in the first 6 weeks of life in intervention vs. control groups, adjusting for pre-trial imbalances.
4. **Were factors related to ACS administration (such as the number of doses and the time between ACS administration and delivery) or the maternal conditions at administration associated with neonatal mortality in the intervention group?** In ACT intervention clusters, health providers were trained to identify pregnant women before 36 weeks’ gestation at risk of preterm birth (i.e., with signs of labor, preterm premature rupture of membranes, pre-eclampsia or eclampsia, or obstetric hemorrhage) and to administer one course of dexamethasone every 12 h. The main aim of this observational analysis was to assess whether factors related to ACS administration were associated with neonatal death. To assess this, we compared the frequency of the factors related to ACS administration between the neonatal deaths and those who survived to 28 days, among livebirths whose mothers were identified as high risk for preterm birth and received ACS in the intervention clusters. We focused on the subgroup of term babies (≥37 weeks gestation), as the harmful effect was primarily in this group, and to reduce the confounding effect of gestational age in the comparison between neonatal deaths and survivors.

The definitions for variables constructed from the study data forms for these analyses are provided in Appendix 1. Unless otherwise noted, the remaining variables are defined as collected on the study forms.

#### Statistical analyses

Generalized linear models were used to assess differences between groups and to develop point and interval estimates for relative risk (RR) of the outcome of interest. Models were log binomial when possible; otherwise Poisson models were utilized. Generalized estimating equations were used to account for the correlation of outcomes within cluster to develop appropriate confidence intervals. In general, models included adjustment for randomization strata except that research site, rather than strata, was included in models assessing differences in the intervention group only (research question 4). For question 3, the proportions of infants with pSBI in clusters assigned to receive the intervention and in control clusters were compared during the pretrial period and during the trial period. Relative risks for pSBI during the trial period were estimated with adjustment for randomization strata only and again with adjustment for both strata and the pretrial proportions of pSBI at the cluster level. All tests were performed at a nominal significance level of α = 0.05. Due to the exploratory nature of the analyses, no correction was made for multiple comparisons. Additional statistical methods are noted under the results of each question, as needed. Analyses were done by RTI International with SAS versions 9.3 and 9.4 (SAS Institute, Cary, NC, USA).

#### Approvals

The ACT trial was reviewed and approved by the ethics committees at each site, the World Health Organization and the *Eunice Kennedy Shriver* National Institute of Child Health and Human Development (NICHD). An independent data monitoring committee appointed by NICHD reviewed the progress of the trial, as specified in the protocol. All women provided informed consent prior to enrollment.

#### Role of the funding source

Staff from the funder (NICHD) had input into study questions and data interpretation and reviewed and approved the report. However, the authors’ views do not necessarily represent those of the NICHD. The authors had access to all the data in the study upon request and all reviewed and approved the paper prior to submission.

## Results

**Fig. 1:**
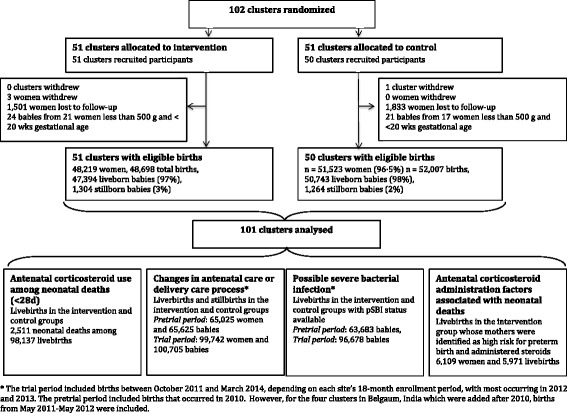
Trial profile and analyses

The study populations for each analysis are shown in Fig. 1. Overall, the population included 48,219 women and 48,698 babies (47,394 live births) in the ACT intervention group and 51,523 women and 52,007 babies (50,743 live births) in the control group. At 28 days, there were 1300 neonatal deaths (27.4/1000 livebirths) in the intervention group and 1211 (23.9/1000 livebirths) in the control group. When we limited analyses to the newborns whose mothers received ACS in the intervention group, the population included 6109 women and 6257 babies (of whom 5971 were livebirths).

### Antenatal corticosteroids as a cause of neonatal mortality

The ACS exposure among the infants who died up to 28 days was 29 % in the intervention group compared to 6 % in the control group. Among infants who were ≥25^th^ percentile for birthweight and died up to 28 days, the ACS exposure was 11 % and 2 % in the intervention and control groups, respectively.

### Did the intervention change the process of care compared to the control group?

There were no substantial differences in antenatal care attendance rates between the groups. The proportion of women screened for syphilis or HIV was also similar between groups, as were the use of preventive interventions such as tetanus vaccine and prenatal vitamins or iron (Table 1). Fewer women in the intervention group were delivered by a physician or in hospital during the trial period. However, similar differences in delivery location and attendant existed during the pretrial period, as well. A smaller proportion of women in the intervention group delivered at facilities with C-section capabilities. No substantial differences were observed between the proportions of women in the intervention and control groups who delivered in facilities with all care capabilities. Additionally, the mode of delivery was similar in the groups with 15 % cesarean section during the trial period. Intervention and control groups also had similar use of new gloves (approximately 94 %), and a clean razor to cut the umbilical cord (99 %), as available measures of quality of care. The proportion of babies that received resuscitation was slightly lower in the intervention than in the control group (7.1 % vs 7.7 %), but a similar trend was observed in the pre-trial period (4.7 % vs 5.7 %).

**Table 1:** Factors related to process of care by ACT treatment group among all births (SB + LB)

Characteristic	Pretrial		Trial	
	Treatment	Control	Treatment	Control
Deliveries, N	30,492	34,533	48,219	51,523
Antenatal Care				
Any antenatal care	28,743 (94.4)	32,801 (95.1)		
Number of antenatal visits	*Data not collected during pretrial period*		45,374	48,052
0			1216 (2.7)	1111 (2.3)
>3			24,663 (54.4)	25,491 (53.0)
Trimester of 1st antenatal visit			43,980	46,632
1st			22,196 (50.5)	24,801 (53.2)
2nd			14,648 (33.3)	14,059 (30.1)
3rd			7136 (16.2)	7772 (16.7)
Administration of diagnosis tests or preventive care				
Syphilis or HIV test	21,944/30,435 (72.1)	24,071/34,446 (69.9)	37,975/47,961 (79.2)	40,343/51,185 (78.8)
Tetanus toxoid vaccine	26,892/30,422 (88.4)	30,467/34,478 (88.4)	40,313/47,980 (84.0)	44,453/51,219 (86.8)
Prenatal vitamin/iron	27,706/30,405 (91.1)	30,829/34,472 (89.4)	44,321/47,952 (92.4)	47,212/51,191 (92.2)
Delivery care				
Delivery attendant	30,490	34,531	48,215	51,519
Physician	10,305 (33.8)	12,709 (36.8)	19,122 (39.7)	23,233 (45.1)
Nurse/nurse midwife/LHW	10,348 (33.9)	10,094 (29.2)	18,166 (37.7)	15,366 (29.8)
TBA/Family/Unattended	9837 (32.2)	11,728 (34.0)	10,927 (22.7)	12,920 (25.1)
Delivery location	30,482	34,494	48,217	51,519
Hospital	12,013 (39.4)	15,008 (43.5)	23,798 (49.4)	27,345 (53.1)
Clinic	8486 (27.8)	7619 (22.1)	13,593 (28.2)	11,675 (22.7)
Home/Other	9983 (32.8)	11,867 (34.4)	10,826 (22.5)	12,499 (24.3)
C-section	3001 (9.8)	3279 (9.5)	7133/48,218 (14.8)	7655/51,520 (14.9)
Use of new gloves	28,391/30,240 (93.9)	32,262/34,140 (94.5)	44,932/47,860 (93.9)	48,587/51,095 (95.1)
Use of clean razor	27,685/30,263 (91.5)	31,707/34,181 (92.8)	46,963/47,248 (99.4)	49,846/50,261 (99.2)
Births at facility with c-section capabilities	9122/27,831 (32.8)	10,859/31,783 (34.2)	17,802/43,072 (41.3)	21,038/45,190 (46.6)
Births at facility with C-section and neonatal care capabilities^a^	4134/27,831 (14.9)	4590/31,783 (14.4)	11,138/43,072 (25.9)	12,290/45,190 (27.2)
Babies, N	30,762	34,863	48,698	52,007
Babies receiving resuscitation	1451/30,757 (4.7)	2001/34,861 (5.7)	3429/48,535 (7.1)	3973/51,871 (7.7)

^a^Neonatal care capabilities include bag and mask, and oxygen or mechanical ventilation

### Did the intervention increase risk of neonatal possible severe bacterial infection (pSBI)?

During the pretrial period, 12.4 % of live born infants in the ACT intervention group versus 14.2 % of infants in control clusters had pSBI (Table 2). Risk of pSBI was not significantly different for infants during the pretrial period in intervention versus control clusters overall [adjusted RR: 0.95 (0.75–1.21), *p* = 0.68]. Similarly, during the trial period risk of pSBI was not significantly different for infants in intervention compared to control clusters overall after adjustment for pretrial rates [14.8 % vs. 13.9 %, adjusted RR: 1.05 (0.92–1.20), *p* = 0.44]. Among infants with birth weight <25^th^ percentile, the relative risk during the trial period was similar to that in the overall population [adjusted RR: 1.03 (0.92–1.15), *p* = 0.63]. However, in infants with birth weight ≥25^th^ percentile we observed a marginally significant 15 % increase in the risk of pSBI [adjusted RR: 1.15 (0.98–1.35), *p* = 0.08].

**Table 2:** pSBI and pSBI plus death in the first 6 weeks of life among live born infants in ACT intervention clusters during the pretrial and trial periods

pSBI				
Characteristic	Intervention	Control	Adjusted RR (95 % CI) of pSBI Intervention vs. Control^a^	Adjusted RR (95 % CI) of pSBI Intervention vs. Control w/adjustment for pretrial pSBI %^a^
Pretrial, N	29,783	33,900		
pSBI, *n* (%)	3702 (12.4)	4814 (14.2)	0.95 (0.75–1.21)	
ACT period, N	46,688	49,990		
pSBI, *n* (%)	6891 (14.8)	6945 (13.9)	1.01 (0.89–1.14)	1.05 (0.92–1.20)
< 25^th^ P^c^	2718/10,479 (25.9)	2818/10,726 (26.3)	0.99 (0.89–1.11)	1.03 (0.92–1.15)
≥ 25^th^ P	4058/36,007 (11.3)	4015/39,030 (10.3)	1.10 (0.95–1.28)	1.15 (0.98–1.35)
pSBI and Death				
Characteristic	Intervention	Control	Adjusted RR (95 % CI) of pSBI & death Intervention vs. Control^a^	Adjusted RR (95 % CI) of pSBI & death Intervention vs. Control w/adjustment for pretrial pSBI %^a^
Pretrial, N^b^	29,780	33,892		
Had pSBI and died, n (%)	681 (2.3)	829 (2.4)	0.96 (0.87–1.07)	
ACT period, N	46,688	49,990		
Had pSBI and died, n (%)	1132 (2.4)	1018 (2.0)	**1.16 (1.04**–**1.29)**	**1.17 (1.04**–**1.30)**
< 25^th^ P^c^	627/10,479 (6.0)	601/10,726 (5.6)	1.02 (0.90–1.16)	1.03 (0.90–1.17)
≥ 25^th^ P	405/36,007 (1.1)	317/39,030 (0.8)	**1.36 (1.13**–**1.64)**	**1.36 (1.12**–**1.65)**

^a^Relative risks and confidence intervals from log binomial or poisson models fit to the binary pSBI or pSBI and death outcome that included effects for randomization strata and intervention group, with and without adjustment for pretrial pSBI proportions at the cluster level. Relative risks significantly different from 1.0 are shown in bold

^b^11 infants born in the pretrial period who had pSBI and were missing 6 week status were excluded (three intervention, eight control)

^c^Birth weight percentile was missing for 436 (0.5 %) infants (intervention: 202, control: 234) with missing measured birth weight

The risk of pSBI plus neonatal death did not differ significantly during the pretrial period for the intervention versus control group [2.3 % vs. 2.4 %, adjusted RR: 0.96 (0.87–1.07), *p* = 0.45]. During the trial period, the risk of pSBI and death was increased for infants in the intervention compared to control group overall [2.4 % vs. 2.0 %, adjusted RR: 1.17 (1.04–1.30), *p* = 0.008]. Similarly, among infants with birth weight ≥25^th^ percentile risk of pSBI plus death was increased during the trial period for infants in the intervention compared to control group [adjusted RR: 1.36 (1.12–1.65), *p* = 0.002]. However, among infants with birth weight <25^th^ percentile, no increased risk of pSBI plus death was observed in the intervention group [adjusted RR: 1.03 (0.90–1.17), *p* = 0.67.]

### Which factors related to ACS administration were associated with neonatal deaths?

Factors related to ACS administration in the treatment group among the infants who died compared to those who survived to day 28 are shown in Table 3, by term versus preterm delivery. Overall, infants who died were more likely to have mothers who received ACS within 7 days of delivery compared to those who survived 28 days (58.2 % vs 32.0 %; *p* < 0.0001). They were also more likely to receive fewer doses of dexamethasone; 32.5 % of infants who died received only one dose compared to 20.9 % in those who survived 28 days. Regarding the maternal conditions at the time of ACS administration, those women whose infants died were less likely to have been identified due to signs of preterm labor and more likely to have had hypertension or hemorrhage. Where a woman was identified or received the first dose did not vary substantially for infants who died compared to those who survived. In the group of term babies a similar pattern was observed. Term infants who died were more likely to have mothers who received ACS within 7 days of delivery compared to survivors (26.5 % vs 17.9 %; *p* = 0.0140), and were also more likely to have mothers who had hypertension and less likely to have mothers who were identified due to signs of preterm labor, compared to survivors. However there were no substantial differences in the number of doses received.

**Table 3:** ACS administration characteristics in neonatal deaths <28 days compared to survivors at 28 days by prematurity among those identified by the intervention who received steroids

Characteristic- *n* (%)	Preterm			Term			Total^b^		
	ND <28d	LB, alive at 28d	P^a^	ND < 28d	LB, alive at 28d	P^a^	ND < 28d	LB, alive at 28d	P^a^
Women, *N*	204	1639		100	3672		304	5311	
Time since 1st dose to delivery	203	1633	*	98	3597	*	301	5230	**
Less Than 2 Days	101 (49.8)	765 (46.8)		16 (16.3)	411 (11.4)		117 (38.9)	1176 (22.5)	
2–7 Days	48 (23.6)	263 (16.1)		10 (10.2)	232 (6.4)		58 (19.3)	495 (9.5)	
8–30 Days	26 (12.8)	279 (17.1)		14 (14.3)	808 (22.5)		40 (13.3)	1087 (20.8)	
More than 1 month	28 (13.8)	326 (20.0)		58 (59.2)	2146 (59.7)		86 (28.6)	2472 (47.3)	
Doses of 6 mg Dexamethasone Received	193	1569		99	3626		292	5195	**
1 dose	82 (42.5)	622 (39.6)		13 (13.1)	464 (12.8)		95 (32.5)	1086 (20.9)	
2 doses	16 (8.3)	120 (7.6)		7 (7.1)	141 (3.9)		23 (7.9)	261 (5.0)	
3 doses	7 (3.6)	64 (4.1)		0 (0.0)	68 (1.9)		7 (2.4)	132 (2.5)	
4 doses	88 (45.6)	763 (48.6)		79 (79.8)	2953 (81.4)		167 (57.2)	3716 (71.5)	
Maternal conditions at moment of receiving corticosteroids	204	1639		100	3672		304	5311	
Signs of preterm labor	139 (68.1)	1208 (73.7)	*	74 (74.0)	2910 (79.2)	*	213 (70.1)	4118 (77.5)	*
PPROM	45 (22.1)	390 (23.8)		16 (16.0)	627 (17.1)		61 (20.1)	1017 (19.1)	
Hemorrhage	31 (15.2)	128 (7.8)	**	4 (4.0)	191 (5.2)		35 (11.5)	319 (6.0)	**
Hypertension	32 (15.7)	233 (14.2)		24 (24.0)	531 (14.5)	**	56 (18.4)	764 (14.4)	
Other	5 (2.5)	39 (2.4)		1 (1.0)	128 (3.5)		6 (2.0)	167 (3.1)	
Where was the woman first identified?	204	1639		100	3672		304	5311	
Community level	109 (53.4)	897 (54.7)		72 (72.0)	2322 (63.2)		181 (59.5)	3219 (60.6)	
Primary health care	69 (33.8)	463 (28.2)		22 (22.0)	934 (25.4)		91 (29.9)	1397 (26.3)	
Hospital	26 (12.7)	279 (17.0)		6 (6.0)	416 (11.3)		32 (10.5)	695 (13.1)	
Where was the injection given, N	204	1639		100	3672		304	5311	
1st dose	204	1639		100	3672		304	5311	
Home	32 (15.7)	280 (17.1)		19 (19.0)	820 (22.3)		51 (16.8)	1100 (20.7)	
Health Center	139 (68.1)	950 (58.0)		72 (72.0)	2332 (63.5)		211 (69.4)	3282 (61.8)	
Hospital	33 (16.2)	409 (25.0)		9 (9.0)	520 (14.2)		42 (13.8)	929 (17.5)	
2nd dose	118	974		86	3198		204	4172	
Home	31 (26.3)	317 (32.5)		30 (34.9)	1184 (37.0)		61 (29.9)	1501 (36.0)	
Health Center	50 (42.4)	401 (41.2)		48 (55.8)	1523 (47.6)		98 (48.0)	1924 (46.1)	
Hospital	37 (31.4)	256 (26.3)		8 (9.3)	491 (15.4)		45 (22.1)	747 (17.9)	

^a^ *P*-value for a binary outcome of death are from generalized linear models with generalized estimating equations to estimate parameters while controlling for cluster correlations. All models are also adjusted for clinical site. In a few instances with small cell counts, p-values are calculated from CMH test adjusted for clinical site ‘*’ indicates statistical significance between 0.0001 to <0.05. ‘**’ indicates statistical significance <0.0001, and blank indicates non-significance

^b^Limited to live births with prematurity status available (5615/6109 (92 %) of women in the intervention group who were identified as high risk for preterm birth and received steroids)

## Discussion

In these secondary analyses of the ACT trial, we explored pathways through which the multifaceted intervention may have increased neonatal mortality in the overall population. First, to determine whether ACS or other components of the intervention could have been responsible for the increased neonatal mortality, we observed that babies who died in the intervention group received ACS five times more frequently than those in the control group. On the other hand, the process of care was either not clinically different between groups, or the lower rates of hospital births observed in the intervention group compared to control group were pre-existent. Second, while the 5 % increase observed in risk of pSBI for infants in the intervention group compared to control was not statistically significant, risk of pSBI plus neonatal death was 17 % greater in the intervention group, a statistically significant increase. Finally, exploring which factors of ACS administration were associated with neonatal deaths, we observed that 27 % of term born infants who died received ACS within 7 days of delivery, compared to 18 % of survivors at 28 days. Term babies who died were also more likely to have had mothers identified due to hypertension and less likely due to signs of preterm labor, compared to survivors. Because this is a secondary analysis of a trial not designed to answer these questions, the results should be considered with caution, but may suggest further lines of research.

The difference observed in neonatal deaths receiving ACS between trial groups, plus the lack of differences in the antenatal and delivery health care process attributable to other components of the multifaceted intervention, strengthen the potential role of steroids as a direct cause of the increased mortality. Moreover, the magnitude of the difference (29 % of neonatal deaths with ACS in intervention vs 6 % in control group) is enough to account for the 11 % higher neonatal mortality rate observed in the intervention clusters [12].

Our findings suggest an increased risk of neonatal severe infection associated with the intervention, primarily for infants with birth weight at or above the 25^th^ percentile. These findings should be interpreted cautiously because this outcome was defined as a composite of clinical signs and symptoms reported by health care workers or family members that has shown a moderate degree of misclassification [19]. Nevertheless, the results are consistent with the increase in suspected maternal post-partum infection reported in the primary trial paper associated with the multifaceted intervention [12]. Furthermore, the trend observed in increased risk of pSBI and the stronger significantly increased risk of pSBI plus death among infants in the intervention group at and above the 25th percentile for birthweight, are in agreement with the harmful effect on neonatal mortality also concentrated among those infants reported in the primary trial paper [12], as well as in term infants. Additionally, a systematic review of small ACS trials reporting outcomes in babies who delivered later than 7 days after ACS administration showed a non-significant 59 % increase in the rates of infant proven infection, however based only on one trial [20]. No trials included in the Roberts & Dalziel 2006 Cochrane systematic review reported neonatal infection outcomes in babies born after at least 36 weeks of gestational age [4]. Although this information is far from proving a causal association, neonatal infection should be further investigated as a main outcome in future research studies.

Finally, it is difficult to interpret the observed associations between a shorter time period between ACS administration and delivery and different maternal conditions at identification as high risk patients (more mothers identified due to hypertension and fewer due to signs of preterm labor) in those babies who received ACS and died compared to those receiving ACS and survived, and should be considered cautiously. First, residual confounding is a possible explanation of differences of such moderate magnitude. Although we focused on the subgroup of term babies trying to prevent confounding by gestational age at birth (babies who died were more likely to be preterm than survivors; and babies born preterm are theoretically more likely to have shorter time period between ACS administration and delivery), differences in gestational age between deaths and survivors are still likely among the babies at term. Unfortunately, the poor gestational age ascertainment at the sites precluded stratification by weeks of gestational age. Second, we would have expected that the time period had been longer and not shorter in the deaths than in survivors, based on the observed harmful effect on neonatal mortality concentrated among ≥25th percentile and term babies [12]. Nevertheless, while ACS are not associated with a change in the latency period prior to preterm delivery in high resource environments, the co-morbidities of nutritional deficiencies and pregnancy abnormalities unique to low resource environments could have resulted in the unanticipated outcomes.

## Conclusions

In summary, these secondary analyses suggest that ACS more than other components of the multifaceted intervention may have been involved in the observed increased neonatal mortality, and also in the observed increased risks of potential severe infections reported in this paper. No clear interpretations can be drawn about the characteristics of ACS administration that could have been associated with a higher risk of neonatal death. These interpretations should be considered cautiously and no practical implications can be derived from them. However, they support that further trials are urgently needed to clarify the effectiveness and safety of ACS on neonatal health in low resource settings, and that neonatal infection should be included as a main outcome in such trials.

## Appendix

**Table 4:** Variable definitions

Variable	Definition and Notes
<5^th^ percentile for birthweight status	The less-than-5th-percentile birthweight group (referred to as less-than-5th-percentile infants) was a proxy for preterm birth and, in view of the differences in birthweight distributions across the sites, was established separately for each site on the basis of birthweight data for the pretrial year. Site-specific cutoffs were 2450 g for Argentina, 2400 g for Zambia, 2267 g for Guatemala, 2000 g for Belgaum, India, 2150 g for Pakistan, 2000 g for Nagpur, India, and 2500 g for Kenya. Infants were classified as less than 5th percentile on the basis of measured birthweights. Estimated weights by clinical assessment were used when measured weights were unavailable; those missing both estimated and measured weights were classified as less than 5th percentile (since based on historical data, most of the missing data were for preterm infants).
<25^th^ percentile for birthweight status	We limited this variable to those with measured birthweight. Those with only estimated birthweight or missing birthweight were excluded. We used site-specific cut offs for the 25^th^ percentile for birthweight.
Preterm/term	The baby’s preterm birth status was calculated using gestational age from last menstrual period and estimated due date. Additionally, we classified or reclassified those with measured birth weight (regardless when measured) greater than or equal to the 95^th^ percentile for weight at 36 weeks gestational age (using site-specific cut offs using WHO weight percentiles calculator that gives gestational age specific distributions http://www.who.int/reproductivehealth/topics/best_practices/weight_percentiles_calculator.xls)) and less than 5500 g as term. 4292 (7 %) of births were coded on weight alone and did not have a GA based on EDD or LMP. This variable resulted in a preterm birth percentage of 11 % of the total population (10 % of live births).
Gestational age band	Beginning with the preterm variable above, we coded the gestational age bands. We first determined whether the gestational age was “well dated”. Pregnancies dated from the actual date of delivery, with a date that is unknown/estimated or without a method recorded for determining the delivery date were considered not well dated. Pregnancies dated from LMP, clinical exam, USG were considered well dated. If the method of obtaining the delivery date was based solely on LMP, then we kept the gestational age from LMP. Otherwise, if the method was clinical exam or USG, or a combination of LMP and one of these, then we kept the gestational age from EDD.
Suspected maternal infection	Composite of process outcomes including receipt of antibiotics plus hospital admission or referral, and one or more of the following: receipt of intravenous fluids, surgery, or other treatment related to infection. Additionally, women with postpartum signs and symptoms of severe sepsis with admission to hospital or sepsis as the primary cause of maternal death were included. The definition also included evidence of antepartum or post-partum infection for mothers with infants with a birthweight less than 2500 g. Antepartum infection was defined as: Antepartum hyperthermia ≥38 °C AND (Chorioamnionitis OR Purulent amniotic fluid) OR Postpartum Infection was defined as: Sepsis postpartum OR (postpartum hyperthermia ≥38 °C AND Antibiotics IM or IV AND at least one of the following: surgery site Infection, foul smelling lochia OR any postpartum intervention (Hysterectomy, Curettage/MVA/Evacuation, blood transfusion)).
Neonatal possible severe bacterial infection (pSBI)	Neonatal symptoms occurring during the first 6 weeks of life and reported in the GN Maternal and Newborn Health (MNH) Registry were used to derive estimates of possible severe bacterial infection (pSBI) based on the WHO Young Infants Clinical Signs Study criteria [Young Infants Clinical Signs Study Group 2008] to the extent possible given the information recorded in the registry. The presence or absence of each of the following symptoms was recorded in the registry, with a “yes” response considered consistent with pSBI: breathing problems/difficulty breathing, feeding problems/stopped suckling/feeding, high fever (>38 °C), hypothermia (<35 °C), convulsions, and bleeding/pus-like discharge from umbilicus. Infants who died with cause of death coded “infection” were also counted as having pSBI. Additionally, text fields used to record information about symptoms and diagnoses as well as cause of death were reviewed. Infants with any of the symptoms listed above, or infection, sepsis, possible sepsis, septic conditions (eg, septic rash, septic cord), meningitis, and/or pneumonia in any of the text fields were counted as having pSBI. The number of pSBI episodes and the exact timing of infection could not be determined, as dates of diagnosis were not recorded. Two of the 63,685 livebirths in the ACT pretrial period are missing pSBI status: one infant with neonatal outcome coded as born alive but cause of death indicated fetal demise (Guatemala, control) and one infant with missing delivery date (Pakistan, intervention). Of the 98,137 livebirths in the trial period, 1459 (1.5 %) are missing pSBI status (intervention: 706, control: 753). The majority (1454) were 2013 births in Pakistan with some or all data collected on revised forms that did not include the symptom questions used to define pSBI. Three other infants with neonatal outcome coded as born alive but with cause of death recorded as fetal demise or macerated stillbirth are missing pSBI status (one from Nagpur, two from Guatemala) and two others from Kenya. Some limitations of this definition include the following, We were unable to match YICSS symptoms completely. Some symptoms, especially breathing problems, were non-specific, as they overlap with viruses, malaria, prematurity, etc. There was a lack of confirmatory tests such as blood cultures or chest x-rays and possibly reporting differences between sites.
Location of delivery by availability of delivery and neonatal care at the facility	We limited the data to births that either occurred at home or at a facility that is generally used by the women living in the MNH clusters. This included facilities that are physically located within the geographic boundaries of the clusters and facilities that are outside the cluster, but are regularly utilized by the women. For each facility, we determined whether or not each of the following services had been provided to at least two people during the course of the trial: c-section, bag and mask, and oxygen or mechanical ventilation. If so, then the facility was coded as having the service. If not, the facility was coded as not having the service. We could then determine whether each birth occurred at a facility with these services available. The variable has the following levels: • Home birth • Delivered at hospital or clinic with none of the maternal/neonatal capabilities • Delivered at hospital or clinic with some, but not all maternal/neonatal capabilities • Delivered at hospital or clinic with all maternal/neonatal capabilities
